# Medical students’ and public obstetric health care workers’ knowledge of the Saving Mothers campaign 

**DOI:** 10.4102/phcfm.v3i1.184

**Published:** 2011-02-28

**Authors:** Almereau Prollius, Gina Joubert, Adelien du Toit, Susan Joubert, Tarina Lourens, Johanna J. Steenkamp

**Affiliations:** 1 Department of Obstetrics and Gynaecology, University of the Free State, South Africa; 2 Department of Biostatistics, University of the Free State, South Africa; 3 School of Medicine, University of the Free State, South Africa

## Abstract

Maternal mortality in South Africa has been receiving attention since it became notifiable in 1997. The ’big five’ causes of maternal mortality are non-pregnancy-related infections (mainly HIV), complications of hypertension during pregnancy, obstetric haemorrhage, pregnancy-related sepsis and pre-existing medical conditions. In many cases in which women die during pregnancy or childbirth, avoidable health worker-related factors can be identified. This study assessed the knowledge of different levels of medical students and health care workers at public health obstetric facilities in Bloemfontein concerning the Saving Mothers campaign. The self-administered, test-like questionnaire was completed by senior medical students, interns and obstetric personnel (nurses or midwives). Interns obtained the highest median score (48%) for the questionnaire, while nurses obtained a median score of 31%. The results strongly suggest that training specific to the Saving Mothers campaign is urgently required across all levels of health care personnel.

## To the Editor

In recognition of the need to reduce maternal mortality in South Africa, deaths during pregnancy, childbirth and the puerperium were made notifiable on 01 October 1997. This was enacted in the National Policy Health Act, Number 116 of 1990.^1^ The Minister of Health appointed a National Committee on Confidential Enquiries into Maternal Deaths, who were responsible for the confidential enquiry into maternal mortality in South Africa. In doing so, the committee developed a reporting system for maternal deaths from which the Saving Mothers reports were compiled. These reports identified the five main causes of maternal deaths and included recommendations to reduce maternal mortality.^2^ The ‘big five’ causes of death during 2001 were non-pregnancy-related infections (mainly HIV), complications of hypertension in pregnancy, obstetric haemorrhage, pregnancy-related sepsis, and pre-existing medical conditions. In more than half of the cases in which a mother died (56.8%), avoidable health worker-related factors in the management of the event were identified. This was most significant at the primary level, with avoidable factors at some point in the woman’s care in almost three-quarters of cases in which there was sufficient information to make the case assessable. The figure dropped to two-thirds for secondary level care and to just below 50% for tertiary level care.^3^ 

The aim of this study was to assess the knowledge of different levels of medical students from the University of the Free State and health care workers at public health obstetric facilities in Bloemfontein regarding the Saving Mothers guidelines and recommendations (published in 1999^4^ and 2003^3^ respectively). Facilities included Universitas Academic Hospital, Pelonomi Regional Hospital, Heidedal Clinic and the Mangaung University Community Partnership Programme (MUCPP) clinic. The study was conducted between August and November 2004.

## Method>

A descriptive study was performed across 393 respondents, including fourth- and fifth-year medical students from the five-year curriculum (n = 109 and n = 71, respectively) and sixth-year students from the six-year curriculum (n = 102) at the University of the Free State (UFS), interns (n = 56) and the obstetric personnel (student nurses, nurses and midwives) at primary health care facilities (n = 55). The entire population was targeted for inclusion in the study. The only exclusions were those who did not give permission to participate, could not be reached at the time of the study, and were not capable of completing an Afrikaans or English questionnaire. The instrument used for this study was a self-administered, test-like questionnaire containing mainly open-ended questions regarding hypertension during pregnancy and labour, obstetric haemorrhage, abortion and pregnancy-related sepsis, and HIV and non-pregnancy-related infections. The questionnaire also included a permission slip and items concerning demographic information. As far as possible, at least one researcher was present during the completion of the questionnaires. A pilot study was conducted among third-year medical students to ensure that the questionnaire was understandable and easily administered. The Ethics Committee of the Faculty of Health Sciences, UFS, approved the study.

Permission to distribute the questionnaires amongst the obstetric personnel at the hospitals and clinics was obtained from either a senior nurse or a matron at the facilities. The questionnaires were distributed among day-shift personnel on a date subsequently agreed on. Permission was obtained from each of three lecturers who presented a lecture to the fourth-, fifth- and sixth-year students to hand out the questionnaires during their classes. The questionnaires were collected at the end of the lectures. An intern distributed questionnaires among interns and collected them again. Responses were coded as correct or incorrect according to a detailed memorandum.

## Results

The response rates for the different groups were as follows: nurses = 43%, interns = 58%, fourth-year students = 37%, fifth-year students = 49%, and sixth-year students = 78%. The median correct answers achieved by each group were as follows: nurses = 31%, interns = 48%, fourth-year students = 40%, fifth-year students = 35% and sixth-year students = 46%. Table 1 compares the results obtained by the different groups, stating each question and the percentages of respondents having the answers either all correct or all incorrect. 

**TABLE 1:**
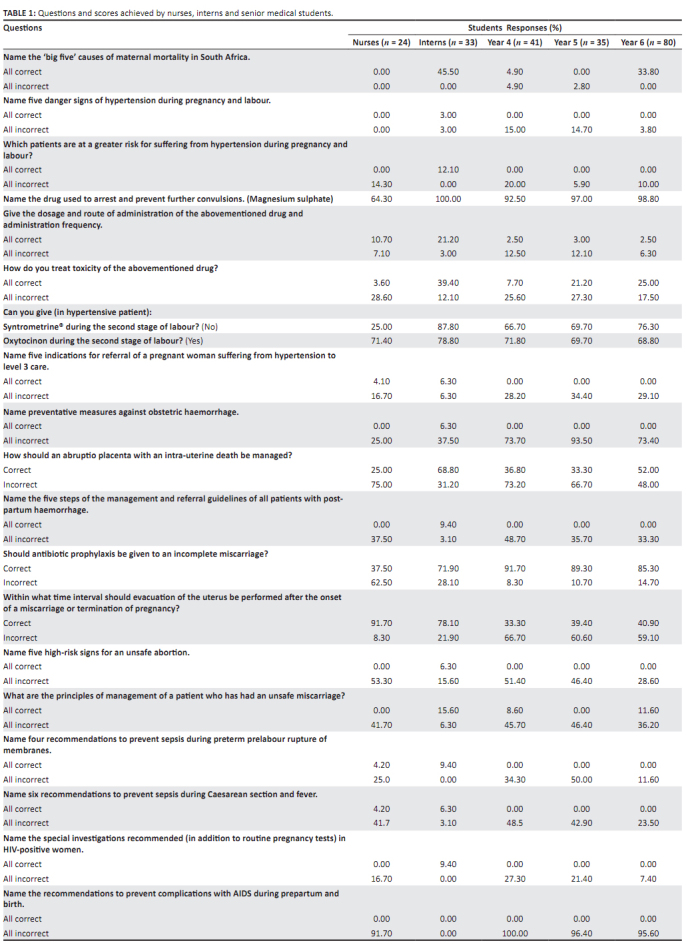
Questions and scores achieved by nurses, interns and senior medical students

## Discussion

Low response rates occurred in some of the groups. In view of this limitation, the results showed that the responding interns had the most knowledge while the responding nurses had the least. Among the students, the responding sixth-years did the best. The evaluation was done among participants working at primary (Heidedal and MUCPP), secondary (Pelonomi) and tertiary (Universitas) level health care facilities. However, participants were not selected or categorised according to the specific level of the facility in which they were working at the time of the study, and it could therefore be construed that no level of care seems exempt from poor performance. Interns’ median score of less than 50% was also a matter of concern. The intern group received their training at various universities throughout the country, and this observation suggests that their lack of knowledge was probably not a regional problem confined only to the Free State. The different levels of health care workers did not have enough knowledge about the campaign and its guidelines to reduce maternal mortality rates in Bloemfontein.

We recommend that all levels of health care workers should undergo specific Saving Mothers guideline training. It should be incorporated in all the health care workers’ training through compulsory lectures. The Saving Mothers guidelines should also be published in a pocketbook format to which health care workers can readily refer in any situation. This was implemented at the Bloemfontein Hospital Complex for interns in 2005. Enough provision must be made for obstetrics in the final year of the five-year curriculum. The same Saving Mothers questionnaire should be re-administered after a predetermined period of using the pocketbook and receiving compulsory lectures on the matter to evaluate whether the knowledge of health care workers has improved and whether their knowledge is sufficient.
